# Two competing cryoballoon technologies for single shot pulmonary vein isolation: first experiences with the novel system

**DOI:** 10.31083/j.rcm2304118

**Published:** 2022-04-01

**Authors:** Guram Imnadze, Thomas Fink, Mustapha El Hamriti, Leonard Bergau, Martin Braun, Moneeb Khalaph, Vanessa Sciacca, Khuraman Isgandarova, Denise Guckel, Christian Sohns, Philipp Sommer

**Affiliations:** ^1^Clinic for Electrophysiology, Herz- und Diabeteszentrum NRW, Ruhr-University Bochum, 32545 Bad Oeynhausen, NRW, Germany

**Keywords:** atrial fibrillation, pulmonary vein isolation, cryoballoon

## Abstract

Following its introduction into clinical practice, the cryoballoon (CB) has proved to be an alternative for pulmonary vein isolation (PVI) in patients with paroxysmal and persistent atrial fibrillation (AF). In comparison with the standard radiofrequency procedure, the CB method results in a shorter procedure time and learning curve as well as a higher degree of reproducibility. A new cryoballoon (NCB) was recently introduced on the market. In this review, we addressed the following questions: Is the new system technically similar to the previous one? Is there a difference in terms of periprocedural parameters? Are acute success and complication rates similar? Is the learning curve different?

## 1. Introduction

Since 2007, cryoballoon (CB) ablation has become an alternative to the radiofrequency technique for achieving pulmonary vein isolation (PVI) in the treatment of patients with symptomatic atrial fibrillation (AF). This rapid and reproducible technique has developed into a first-line therapy for PVI [1, 2]. Compared to radiofrequency ablation; noninferiority for efficacy and safety of the cryoballoon system has been reported in a number of studies [1, 3, 4, 5, 6, 7, 8]. In comparison to the cryoballoon with early generation devices, the fourth-generation cryoballoon offers improvements such as shorter duration of the procedure, shorter balloon-in-body time, a shorter learning curve and a higher reproducibility rate [9, 10, 11]. In these studies, PVI was performed using standard cryoballoons (SCB). Approximately one million procedures have been performed using this technique worldwide (AFA-Pro; Medtronic, Minneapolis, MN, USA). Recently, a new cryoballoon (NCB) technology (POLARxTM; Boston Scientific, Marlborough, MA, USA) was introduced onto the market [12]. While SCB has been present in several generations, only the first generation of the NCB is currently available on the market.

## 2. Methods and objectives

This review is based on all available reports where the two competing cryoballoon technologies were evaluated. The search was conducted via PubMed and involved the following keywords: (“Cryoballoon” OR (“Polarx” or “Arctic Front”) AND “Pulmonary vein isolation” OR “Atrial fibrillation”). Since NCB was only commercially available since 2020, the analysis was limited to publications from 2019 to 2021.

We analyzed only comparative studies between two cryoballoon systems. To the best of our knowledge, the six articles which are included in this review article are the only available articles published on this topic so far.

We addressed the following questions: Is the new system technically similar to the previous one? Is there a difference in terms of periprocedural parameters (procedural time, fluoroscopy time, left atrial dwell time, minimal temperature, and time to isolation effect)? Are acute success and complication rates similar? Is the learning curve different?

## 3. Technical aspects

**Fig. 1.**
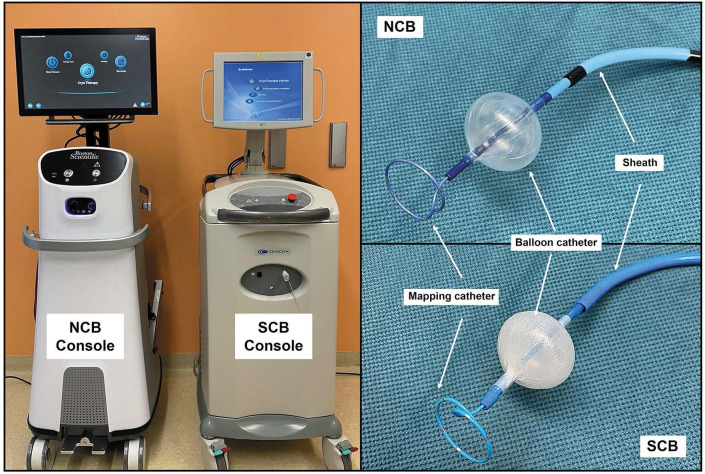
**New and standard cryoballoon technique equipment**. Left — Two consoles. Right upper — The sheath, Cryoballoon catheter and multipolar diagnostic catheter for the NCB technique. Right lower — The sheath, Cryoballoon catheter and multipolar diagnostic catheter for the SCB technique. NCB, new cryoballoon; SCB, standard cryoballoon.

The new system, similar to the SCB model, has several components: console, sheath, balloon catheter and a lasso-shaped multipolar diagnostic catheter (Fig. 1). The technical parameters of both systems are presented in Table 1.

**Table 1.** **Technical characteristics of both system**.

General specifications	SCB	NCB
Sheath diameter (F)	12	12.7
Sheath outer diameter (F)	15	15.9
Radiopaque marker proximal to the tip (mm)	5	2.5
Ballon size (mm)	23 or 28	28
Balloon shaft diamater (F)	10.5	11.8
Balloon tip length (mm)	8	5 or 12
N2O injection	8-hole coil	8-hole coil
N2O fluid flow during freeze (sccm)	7200	7800
Pressure during freeze (psi)	530–600	<525 constant
Location of injection coil from pole of balloon (mm)	3.5	2.5
Location of thermocouple (TC) from coil (mm)	15	18
Location of gas outflow proximal of TC (mm)	10	5
Phrenic nerve palsy controll	CMAP (not integrated/not quantitative)	DMS (integrated/quantitative)
Console register procedural data	no	yes
Console operation autonomicaly	no	yes

SCB, standard cryoballoon; NCB, new cryoballoon; CMAP, compound motor action potential; DMS, diaphragm movement sensor; TC, thermocouple.

## 4. The sheath 

Both cryoballoon systems use steerable sheaths to introduce and maneuver the system inside the left atrium. Although the tools and procedural workflow for both systems are similar, they differ in their handling, as reported in a number of studies [13, 14]. The sheath for the NCM system is 1 Fr larger, but due to its more gradual taper from the dilator to the sheath, it tends to more easily cross the septum. Moreover, the sheath and the balloon shaft in the NCB system are more flexible and softer [13]. The NCB sheath has a radiopaque marker 2.5 mm proximal to the sheath tip; while the SCB sheath radiopaque marker is placed 5 mm proximal to the tip. However, the NCB sheath is delivered without a stopcock [14].

## 5. Balloon catheter

There are some differences in the balloon technologies between the two systems.

The SCB inflation pressure is low. Following the initiation of the ablation, the pressure increases up to six times, which makes the cryoballoon more rigid and slightly increases the size of the CB. Unlike the SCB, the inflation pressure of the NCB remains consistently low during the entire ablation. Therefore, the NCB does not increase in size after the initiation of the ablation. A complete occlusion is required before commencing the freeze of the NCB [15]. However, no difference was reported in the magnitude of PV occlusion between the two systems [10]. It is hypothesized that the compliant nature of the NCB balloon promotes a more antral lesion which might lead to enhanced tissue ablation [15]. Although the location of the thermocouple is similar in both systems, the possibility that the more compliant NCB balloon may bring the thermocouple closer to the cooling area cannot be excluded; since lower balloon temperatures have been documented as compared to the SCB [10].

Previous studies have shown that the balloon thawing time is one of the most reliable biophysical markers of a durable PVI. A longer thawing time represents not only colder; but also a more effective CB application and is believed to promote additional cellular injury [16, 17, 18]. Yap *et al*. [10] showed that the NCB system has a longer thawing time than the SCB. Further investigation is needed to determine whether this difference translates into a higher rate of durable PVI.

## 6. Multipolar diagnostic catheter 

The mapping catheters are also similar in both systems, but it has been observed that there is a higher rate of real-time visualization utilizing the NCB mapping catheter. Time to isolation (TTI) was recorded in a higher percentage of pulmonary veins (PVs) with the NCB than with the SCB (93.1% vs. 79.6%). One of the explanations for this difference could be the shorter distal tip of the NCB (5 mm) in comparison to the SCB (8 mm), which helps to bring the circular mapping catheter more proximal to the pulmonary vein ostium [10].

Another potential reason for the signal quality difference could be the fact that the NCB mapping catheter is manufactured with one continuous length of nitinol wire from the connector to the distal hoop. The mapping catheter of the SCB uses mechanical joints with stainless steel core wire. Furthermore, the NCB mapping catheter insulates both the electrode signal wires and the core wire, whereas the SCB mapping catheter only insulates the electrode signal wires [19]. However, it has been reported that the NCB multipolar mapping catheter is somewhat less stiff and may provide less support [15].

## 7. Console

The NCB console is generally more modern. The pedal is used to inflate/deflate the balloon and to initiate/stop cryo-energy delivery. This option helps the operator to perform the procedure autonomically without assistance. In the upcoming version, the operator can also manage the procedure using a sterile remote control replacing the functions of the foot pedal. The built-in diaphragm movement sensor (DMS) allows for live quantitative assessment of phrenic nerve palsy. It triggers a red warning sign if a reduction in diaphragm contraction is detected and allows the operator to stop the cryo-energy delivery earlier.

## 8. Procedural data

In the published articles, only a limited number of patients (around 50) were analyzed in each NCB and SCB group. Despite the limited number of patients, some technical differences were evident. Baseline characteristics such as age, gender, body mass index, congestive heart failure, hypertension, coronary artery disease, diabetes mellitus and a history of stroke or TIA were similar in both groups in all studies. The percentage of patients with paroxysmal AF was also similar between the two cohorts in all publications. A time-to-isolation (TTI) guided ablation protocol was used in all studies.

The total procedure time as well as left atrial (LA) dwell time was statistically lower in the SCB group in the majority of the studies (Table 2, Ref. [8, 10, 13, 14, 19, 20]) . The fluoroscopy time trended to be lower in the SCB group in all but one report. In contrast to the other studies, Tilz *et al*. [19] reported lower fluoroscopy and total procedural time in favor of the NCB. The amount of contrast agent was not described in all studies, but Yap *et al*. [10] and Moser *et al*. [20] found that it was lower in the SCB group than in the NCB group.

**Table 2.** **Periprocedural characteristics of both systems according to all available publications**.

Author	Patients n.		Procedure time min.			LA dwell time min.			Fluoroscopy time min.			Contrast agent mL.		
	SCB	NCB	SCB	NCB	*p* value	SCB	NCB	*p* value	SCB	NCB	*p* value	SCB	NCB	*p* value
Creta *et al*. [8]	40	40	60	60	0.12	35	39	<0.01	3.3	5.2	0.07	x	x	x
Kochi *et al*. [13]	50	20	60	90	<0.001	x	x	x	13.7	15	0.29	x	x	x
Tilz *et al*. [19]	25	25	55	45	0.06	x	x	x	12	8	0.01	70	60	0.84
Yap *et al*. [10]	53	57	67	81	<0.001	35	51	<0.001	10.8	14	0.14	40	50	0.002
Knecht *et al*. [14]	40	40	65	84	0.003	47	57	0.05	20	25	0.08	x	x	x
Moser *et al*. [20]	50	50	62	80	<0.001	x	x	x	11	17	<0.001	60	70	0.015

SCB, standard cryoballoon; NCB, new cryoballoon; LA, left atrium.

## 9. Minimal temperature 

Balloon temperatures are dependent on multiple factors such as balloon-to-PV size ratio, balloon position and ipsilateral PV blood flow. In all studies, it was shown that the nadir temperature in the NCB cohort was statistically lower than in the SCB group (Table 3, Ref. [8, 10, 13, 14, 19, 20]). The NCB achieves lower balloon nadir temperatures faster than the SCB. However, in contrast to SCB, in NCB cooling rates from –30 ^∘^C or –40 ^∘^C were not associated with acute PVI. The question is if this difference allows the new system to create faster and deeper lesions? Or given that both balloon catheters are of similar construction, how can we explain such a difference in measured temperatures [15]?

**Table 3.** **Minimal temperature differences according to all available publications**.

Author	Min. Temp. LSPV			Min. Temp. LIPV			Min. Tem. RSPV			Min. Temp. RIPV		
	SCB	NCB	*p* value	SCB	NCB	*p* value	SCB	NCB	*p* value	SCB	NCB	*p* value
Creta *et al*. [8]	47.3	59.0	<0.001	48.3	54.4	<0.001	50.6	58.4	<0.001	48.6	56.6	<0.001
Kochi *et al*. [13]	52	35	<0.001	47	32	0.001	40	33	0.24	42	32	0.001
Tilz *et al*. [19]	49	61	<0.001	48	55	<0.001	53	55	0.01	48	56	<0.001
Yap *et al*. [10]	46	55	<0.001	44	54	<0.001	52	58	<0.001	50	55	<0.001
Knecht *et al*. [14]	48	61	<0.001	44	56	<0.001	47	60	<0.001	47	59	<0.001
Moser *et al*. [20]	49	62	<0.001	46	58	<0.001	53	62	<0.001	47	60	<0.001

SCB, standard cryoballoon; NCB, new cryoballoon; LSPV, left superior pulmonary vein; LIPV, left inferior pulmonary vein; RSPV, right superior pulmonary vein; RIPV, right inferior pulmonary vein.

It was reported that the median lowest temperature and temperature during the vein isolation was approximately 10 ^∘^C lower in the NCB group [13]. The constant pressure in the NCB was described as the main difference in one of the initial studies [10, 14].

Knecht *et al*. [14] removed the layers of both balloons and carefully inspected the cooling technology of the catheters. They identified small differences in catheter design (thermocouple, gaseous injection and outflow coil positions) between the two CB systems and suggested that it is the most likely cause for the lower recorded temperature in the NCB system [14]. Another theory is that the NCB has a higher compliance which results in a movement of the thermocouple towards the distal tip where the main source of the cooling is and results in lower temperature measurements. However, the authors did not observe a higher degree of balloon deformation of the NCB compared to the SCB when positioning at the PV ostium [14]. Moreover, insulating capabilities of the double-layer CB material might also be different and can play a role in temperature differences, but this aspect has not yet been studied.

Knecht *et al*. [21] also studied the nadir cryo-balloon temperatures of the freezing cycles of both CBs in a water bath and documented that the difference (–12 ^∘^C lower in the NCB group) was similar to that seen in the *in vivo *study. It was previously shown that with the SCB, after 180 s of application, the local freezing capabilities can be reached beyond the equator of the balloon, which has the potential to impact outcomes [21]. Local ice formation after 180 s application to or beyond the equator of the balloon to the proximal hemisphere could be observed in all cases of the SCB and only in 67% for the NCB. The consistent coverage of the distal hemisphere up to the balloon equator and beyond was documented only with the SCB [14].

Several studies with the SCB system sought to understand the relationship between target temperatures and safety margins; however, this has not yet been studied in the NCB system [15].

## 10. Time to isolation (TTI)

Previous studies have shown that the TTI is the most important predictor of durable PV isolation. TTI effect less than or equal to 60 s is the targeted time during CB ablation in clinical practice [18, 22, 23, 24, 25, 26]. TTI was comparable between the two systems in all studies, despite lower balloon temperatures at TTI with the NCB system [10, 13, 14, 15]. Based on TTI effect analyses, there was no statistical difference between these two cohorts (Table 4, Ref. [8, 10, 13, 14, 19, 20]). Furthermore, there was a trend toward even less TTI effect in the SCB group in most studies. Therefore, the lower temperature is not predictive of a stronger effect when comparing the two systems. Interestingly, the troponin level after ablation was also not different between both groups, thereby indicating a similar degree of tissue damage. The investigators theorized that inside the atrial tissue, the temperature does not differ between both groups and that differences in temperature may be due to different methods of measurement [13].

**Table 4.** **Time to Isolation (TTI) effect according to all available publications**.

Author	TTI LSPV			TTI LIPV			TTI RSPV			TTI RIPV		
	SCB	NCB	*p* value	SCB	NCB	*p* value	SCB	NCB	*p* value	SCB	NCB	*p* value
Creta *et al*. [8]	45	52	0.40	74	54	0.55	36	91	0.06	37	73	0.42
Kochi *et al*. [13]	39	44	0.25	33.5	35.5	0.44	29	32	0.36	30	31	0.61
Tilz *et al*. [19]	50	37	0.23	25	35	0.39	30	40	0.10	40	51	0.43
Yap *et al*. [10]	43*	45*	0.44	* only mean TTI was presented								
Knecht *et al*. [14]	45	53	0.13	47	55	0.56	52	45	0.50	62	55	0.64
Moser *et al*. [20]	32	41	0.11	25	31	0.12	30	29	0.84	38	42	0.47

SCB, standard cryoballoon; NCB, new cryoballoon; LSPV, left superior pulmonary vein; LIPV, left inferior pulmonary vein; RSPV, right superior pulmonary vein; RIPV, right inferior pulmonary vein; TTI, time to isolation.

## 11. Acute success and complications

All articles published to date show a comparable success rate for both groups (Table 5, Ref. [8, 10, 13, 14, 19, 20]). Assaf *et al*. [27] demonstrated in a meta-analysis that patients undergoing the PVI procedure with NCB and SCB systems have a similar acute procedural efficacy [27].

**Table 5.** **Procedural complications and success rates**.

Author	Patients n.		Air embolism		Phrenic nerve palsy		Tamponade		Minor complications		Procedural success %	
	SCB	NCB	SCB	NCB	SCB	NCB	SCB	NCB	SCB	NCB	SCB	NCB
Creta *et al*. [8]	40	40	0	0	1	1	0	1	1	1	100	100
Kochi *et al*. [13]	50	20	0	0	3	0	0	0	1	0	100	100
Tilz *et al*. [19]	25	25	0	1	1	1	0	0	0	0	100	100
Yap *et al*. [10]	53	57	0	1	2	2	0	0	0	1	100	99.5
Knecht *et al*. [14]	40	40	0	1	0	1	0	0	0	0	100	95
Moser *et al*. [20]	50	50	0	2	2	2	0	0	0	0	100	99.5
Total	258	232	0	5 (2.1%)	9 (3.5%)	7 (3%)	0	1 (0.4%)	2 (0.77%)	2 (0.85%)	100	99

SCB, standard cryoballoon; NCB, new cryoballoon.

Information on the average number of cryo-balloon applications and the frequency of PVI at the first application is contradictory. Yap *et al*. [10] showed lower median number of cryoballoon applications in SCB group especially when isolating the right PVs (RSPV *p *< 0.05; RIPV *p *< 0.08) [10]. Similar result was reported from Moser *et al*. [20], the authors described that the procedures conducted with the NCB system required more freeze cycles than procedures conducted with the SCB system (5 [4, 6] vs. 4.5 [4, 5], *p* = 0.002). The difference was mainly driven by more freezing cycles delivered in the RSPV (NCB 1.6 ± 0.9, SCB 1.1 ± 0.3, *p* = 0.001). Frequency of complete isolation of all PVs with one freeze cycle per PV (first-pass) was not significantly different between both groups but tendentially was higher in SCB group (NCB: 28%, SCB: 48% *p* = 0.064) [20]. Knecht *et al*. [14] reported the lower median number of freezes in SCB group (5 vs. 6, *p* = 0.051). The single-shot isolation was achieved in 73% with the NCB compared to 71% in the SCB group (*p* = 0.707) [14]. In contrast, Kochi *et al*. [21] demonstrated that the median number of veins isolated in the first attempt, per patient, was lower in NCB group (3 vs. 4, *p *< 0.001) [13]. Tilz *et al*. [19] demonstrated no difference for the mean total number of freeze cycles per PV until isolation [19].

The long-term success rate in maintaining normal sinus rhythm is the most important outcome of these procedures. However, the number of patients studied in these initial publications is small, which precludes any conclusions that can be made regarding long-term outcomes [28].

There was no difference in complication rates reported between the two groups (Table 5). The cohorts were too small for the assessment of complications, which occurred in only about 0.5–3%. Yap *et al*. [10] presented two phrenic nerve palsies in each group. One patient in the NCB group experienced a moderate left-sided hemiparesis after the procedure which was fully recovered the next day. Creta *et al*. [8] described one cardiac tamponade requiring cardiac surgery in the NCB group, and one patient with temporary phrenic palsy and one patient with a femoral hematoma not requiring any intervention. In the SCB group, complications occurred in two patients; a transient phrenic nerve palsy and hemoptysis on the day after the procedure, which was resolved without further sequelae. No complications were observed in the NCB cohort by Kochi *et al*. [13]; while three temporary phrenic nerve palsies and one pericardial effusion without hemodynamic compromise were described in the SCB group. Knecht *et al*. [14] demonstrated no peri-procedural complications in the SCB group, and one stroke due to air embolism and one transient phrenic nerve palsy in the NCB group. One transient PN palsy occurred in bought groups described from Tilz *et al*. [19]. One transient ST-elevation due to an air embolism was observed in the NCB group. Moser *et al*. [20] also observed an equal proportion of phrenic nerve palsy which occurred once in two patients in both groups. In addition, two cerebral ischemic events occurred in the NCB group. When analyzing the complication rate in general, air embolism in the NCB group seems to be the most important point to be addressed in the future (Table 5). In our institution, we have already observed repetitive air aspiration after the introduction of the NCB sheath in LA (before the introduction of the balloon catheter) in two cases, which was an indication for replacing of the sheath.

There has been special interest concerning the incidence of phrenic nerve palsy in the NCB cohort. Diaphragmatic excursion in the NCB system is assessed by using the Diaphragmatic Movement Sensor (DMS). The sensor uses an accelerometer and provides a relative measure of diaphragmatic excursion.

If the diaphragmatic excursions decreases, the DMS percentage drops. The cutoff for immediate termination of CB application is 65% [10]. Despite the lower temperatures in the NCB groups; a higher incidence of persistent phrenic nerve palsy was not observed [13, 15]. The value of the new DMS system in terms of improvement in the incidence of phrenic nerve palsy needs to be evaluated in larger, randomized studies. It is important to know that using the DMS together with hand palpation; an error in the DMS reading can occur [13].

## 12. Learning curve

No major learning curve was observed for both systems [15]. Despite differences in handling, the similarity of the techniques allows relatively quick mastering of the NCB system. A learning curve effect was demonstrated by Yap *et al*. [10]. The authors described no differences in the procedure times between both platforms when they analyzed the first and the second half of the study cohort [10]. However, a possible explanation for the procedural differences could be the lack of experience with some of the new features and workflow of the NCB, which can occur whenever a new system is introduced into clinical practice [13]. Subjectively, all authors found the NCB system platform easy to handle. Only minor differences were observed when compared to their standard procedural workflow.

The new foot pedal technique may increase operator autonomy, but Creta *et al*. [8] found it may be better to let the lab assistant continue to control some of the console operation functions. They suggested that the foot pedal user interface can lead to decreased catheter lab team interactions. Furthermore, aggressive maneuvers like pull-downs are not recommended by the company; which may alter procedural outcomes [15].

In summary, all studies found that the NCB had similar safety and acute efficacy compared with the SCB. NCB achieved lower temperatures, but TTIs were similar. However, longer procedure and fluoroscopy times were observed in the NCB group. The DMS for phrenic monitoring seems safe and is user-friendly. There is a limited experience with the NCB system so far. Moreover, the new first generation system has only recently emerged on the market, and it should be remembered what a difference was made when the second generation of SCB was released in 2012. Since that time, the SCB improved its design several times and resulted in a progressive reduction in fluoroscopy, ablation and procedural times [13, 29]. We expect the difference in procedural parameters to disappear in the near future between these two techniques.

## 13. Conclusions

The efficacy and safety of NCB are comparable with the SCB. The NCB results in faster cooling rates and lower balloon temperatures, but TTI is similar for both systems, which may be due to minor differences in catheter design. Furthermore, the learning curve seems to be short if there is already experience with the SCB. Future studies with larger sample sizes are necessary to investigate the success rates and safety aspects of the NCB long term.
